# Editorial: New approaches for central nervous system rehabilitation

**DOI:** 10.3389/fneur.2024.1367519

**Published:** 2024-01-31

**Authors:** Pawel Kiper, Agnieszka Guzik, Maurizio Petrarca, Angel Oliva-Pascual-Vaca, Carlos Luque-Moreno

**Affiliations:** ^1^Healthcare Innovation Technology Lab, IRCCS San Camillo Hospital, Venice, Italy; ^2^Department of Physiotherapy, College of Medical Sciences, Institute of Health Sciences, University of Rzeszów, Rzeszów, Poland; ^3^Movement Analysis and Robotics Laboratory (MARlab), Neurorehabilitation Unit, Neurological Science and Neurorehabilitation Area, Bambino Gesù Children's Hospital, IRCCS, Rome, Italy; ^4^Department of Physiotherapy, Faculty of Nursing, Physiotherapy and Podiatry, University of Sevilla, Sevilla, Spain

**Keywords:** neurorehabilitation, robotics, virtual reality, exoskeleton, sensory motor integration, recovery prediction, priming techniques, augmented technique

Neurorehabilitation is a rapidly growing field in motor rehabilitation, which is specifically aimed at restoring neural plasticity of the central nervous system (CNS). The concept of neuroplasticity refers to the ability of the brain to reorganize itself in response to learning or exposure to enriched environments, and it is maintained for the entire human life. Thus, applying specific treatments can be beneficial for people with CNS injuries. The time frame for maximizing the benefits of neuroplasticity is critical, with the plateau observed about 12 weeks after the onset of stroke (1). Thus, it is essential to capitalize on this high level of brain reorganization by providing well-timed and well-designed treatments. A range of approaches has been developed for CNS recovery in acute, subacute, or chronic stages of injury. These approaches include priming or augmented techniques, such as end-effector robots, exoskeletons, or virtual reality, with many being confirmed as effective (2, 3). However, clinical practice still lacks specific indications for which therapy is most effective, for how long should be applied, and for which patient impairments. Therefore, this Research Topic aimed to explore new neurorehabilitative ideas and approaches, modifications of already existing techniques, and identification of research or clinical gaps, including predictive research for treatments and recovery.

There is a growing body of evidence supporting the use of innovative technologies like exoskeletons and/or orthoses (Cho et al.), virtual reality (Bian et al.) (4), and brain-computer interfaces in neurorehabilitation (Carino-Escobar et al.; de Freitas Zanona et al.). These technologies can provide a more immersive and engaging environment for therapy, and some studies have reported significant improvements in motor function and cognitive abilities in patients with CNS injuries (5). In addition to novel intervention techniques, the use of diagnostic techniques that measure cortical activity offers deeper knowledge about motor learning (6) and the changes that these techniques can cause, not only at a functional level, but also in terms of neuroplasticity. However, further research is needed to determine which technologies and interventions are most effective for different patient populations and to develop personalized treatment plans. In addition to innovative technologies, there is a growing interest in innovative approaches to neurorehabilitation, such as peripheral electrical stimulation (Mijic et al.), Tai Chi training (Huang et al.), and audio-luminous biofeedback training for ocular motility (Misawa et al.) that have shown promise in enhancing motor function in patients with CNS injuries. However, the optimal parameters for these interventions and the patient populations that would benefit most from them are still being investigated (Paniagua-Monrobel et al.; Steendam-Oldekamp et al.).

The collection of this Research Topic included ten articles, mostly original, with a two review reports and two case reports. The original research focused on the exploration of diverse neurorehabilitative interventions and their applications across different stages of CNS injury, including acute, subacute, and chronic phases. Researchers aimed to investigate the efficacy of various neurorehabilitative interventions across different stages of CNS injury. Ten studies, spanning diverse research methodologies such as randomized controlled trials, case series, case reports, and literature reviews were, incorporated into this Research Topic.

In the study by Bian et al. authors aimed to evaluate the impact of a non-immersive virtual reality (VR)-based intervention on lower extremity movement in stroke patients, comparing its effectiveness to conventional therapies. The results suggested that the non-immersive VR-based intervention could be a valuable adjunct to conventional physical therapies, potentially augmenting overall treatment efficacy. Despite not exhibiting significant differences from conventional therapies, the intervention demonstrated noteworthy improvements in walking speed, balance, and lower extremity movement. This underscores the potential of non-immersive VR interventions as valuable complements to traditional therapies for enhanced treatment outcomes. In a study by Carino-Escobar et al. researchers explored the effectiveness of an experimental brain-computer interface (BCI) therapy in a 41-year-old COVID-19 patient who experienced a stroke. The patient, lacking traditional stroke risk factors, exhibited notable recovery in upper limb function through the BCI intervention during the chronic stroke stage. This highlights the innovative potential of BCI interventions in achieving significant motor recovery, even in COVID-19-related strokes during the chronic phase. Also, de Freitas Zanona et al. explored the effects of combining BCI with mental practice (MP) and occupational therapy (OT) on activities of daily living (ADL) performance in stroke survivors. Participants were randomized into experimental (BCI, MP, and OT) and control (OT only) groups. The experimental group showed significant improvements in various evaluations, indicating enhanced functional independence and sensorimotor recovery. Notably, the BCI group demonstrated larger effect sizes compared to the control group, suggesting the potential of BCI in promoting ADL performance and social participation in subacute post-stroke survivors. Steendam-Oldekamp et al. assessed the effectiveness of a multidisciplinary in-and-outpatient rehabilitation program for patients with advanced Parkinson's disease (PD) in stabilizing activities of daily living (ADL) and delaying nursing home admission. The intervention group, which underwent a 6-week inpatient program followed by a 2-year outpatient support program, showed significant improvements in ADL functions compared to the control group. After 2 years, 65% of the intervention group continued to live independently at home. The study emphasizes the substantial benefits of intensive rehabilitation for advanced PD patients, highlighting the potential to enhance their quality of life and delay nursing home admission. Study by Cho et al. explored the clinical effects of 3D-printed ankle-foot orthoses (3D-AFOs) on community ambulation in patients with chronic stroke. Three cases were presented, and gait assessments were conducted under various conditions. Following 4 weeks of community ambulation training with 3D-AFOs, improvements were observed in step length, stride width, ankle range of motion, and muscle efficiency during walking and stair ascent. While the training did not significantly impact patient participation, it enhanced ankle muscle strength, balance, gait symmetry, and endurance, and reduced depression. Patients expressed satisfaction with 3D-AFOs, citing their thinness, lightweight, comfort with shoes, and gait adjustability. The study by Huang et al. aimed to assess the impact of 12 weeks of Tai Chi exercise on neuromuscular responses and postural control in elderly individuals with sarcopenia. Sixty participants were randomly assigned to the Tai Chi group or the control group. After the intervention, the Tai Chi group exhibited a significant decrease in neuromuscular response times and overall stability index, indicating improved dynamic posture control and reduced fall risk. The Tai Chi group outperformed the control group in these measures. The simplified Tai Chi protocol proved feasible, safe, and effective for enhancing neuromuscular responses and postural control in elderly sarcopenic individuals, suggesting potential benefits in fall prevention. Misawa et al. conducted prospective pilot study and investigated the effectiveness of biofeedback training (BT) in individuals with homonymous hemianopsia (HH) and brain injury from various etiologies. Participants underwent five weekly BT sessions, leading to improvements in paracentral retinal sensitivity, fixation stability, contrast sensitivity, near vision visual acuity, and reading speed. Overall, the study suggests that BT resulted in encouraging enhancements in visual functions and functional vision for individuals with HH. Further confirmation through larger trials is needed, but the results indicate positive outcomes, particularly in visual ability, visual information, and mobility. Additionally, Paniagua-Monrobel et al. in their observational study, aimed to identify a “preferential patient profile” (PPP) for stroke survivors who may benefit more from early physical therapy (PT) treatment. Analyzing data from 137 individuals with stroke, they found that the PPP for early outpatient PT was a young person with left or bilateral haemorrhagic stroke. The results suggested that direct referral to PT services for this profile could lead to shorter waiting times and potentially greater recovery. The study highlights the importance of establishing a definitive profile through homogenous functional evaluations at the beginning and end of PT treatment. Further research with such evaluations is recommended to refine the PPP for efficient rehabilitation.

In the systematic review conducted by Mijic et al. researchers explored the potential role of peripheral electrical stimulation (PES) in altering somatosensory evoked potentials (SEPs) in both healthy subjects and stroke patients. Despite insufficient evidence confirming SEPs as predictors of rehabilitation prognosis after stroke, a correlation was found between sensory and motor function assessments and changes in SEP components. Notably, PES interventions, particularly when linked to voluntary contractions for specific movements, showed positive relationships with motor function assessments. The study suggests that repetitive, task-oriented treatments enriched with PES could offer a distinct approach in stroke rehabilitation, potentially impacting motor neuroplasticity. However, further randomized controlled trials are needed to validate these findings and determine the utility of SEPs in monitoring the therapeutic effects of PES. Finally, the scoping review conducted by Favetta et al. explored the diffusion of motor control models in post-stroke rehabilitation literature. Authors analyzed 45 studies revealing a lack of clear theoretical bases in most stroke rehabilitation interventions. Only 10 studies explicitly stated the reference theoretical model. The classifications showed 21 studies referring to the robotics motor control model, 12 to self-organization, eight to neuroanatomy, and four to the ecological model. Results indicated a prevalent absence of explicit theoretical frameworks in stroke rehabilitation interventions, emphasizing the need for attention to theoretical underpinnings in designing future experimental approaches for stroke rehabilitation. The study highlights the importance of establishing solid scientific hypotheses on motor control and learning principles to advance rehabilitation as a scientifically-driven process.

The collective insights gained from the studies discussed have significantly advanced our understanding of neurorehabilitation, shedding light on the potential of various interventions informed by motor control and motor learning models. However, the scoping review by Favetta et al. underscored a notable gap in the explicit declaration and application of theoretical frameworks in many neurorehabilitative interventions. This gap highlights the need for greater attention to theoretical underpinnings in the design and reporting of interventions, emphasizing the importance of integrating scientific concepts into clinical trials. Moving forward, future research endeavors in neurorehabilitation should focus on addressing these identified gaps to further refine and expand our understanding of effective therapeutic approaches. It is crucial to explore the underlying mechanisms through which interventions impact neurorecovery, giving the basic for the development of more targeted and personalized rehabilitation strategies. One key avenue for future exploration is the integration of emerging technologies, such as virtual reality, brain-computer interfaces, and 3D printing, into rehabilitation protocols. The studies by Bian et al., Carino-Escobar et al., Cho et al., and de Freitas Zanona et al. demonstrated the potential of these technologies to enhance traditional therapeutic approaches, offering personalized, engaging, and effective interventions. Investigating the optimal ways to integrate these technologies into routine clinical practice and identifying the specific patient populations that may benefit the most will be crucial for the continued advancement of neurorehabilitation.

Additionally, research efforts should delve deeper into the mechanisms of action behind successful interventions. Understanding the neuroplastic changes, both at the structural and functional levels, induced by different therapeutic modalities will provide valuable insights. Advanced neuroimaging techniques, such as functional magnetic resonance imaging (fMRI) and diffusion tensor imaging (DTI), could be employed to unravel the intricate processes occurring within the central nervous system during recovery. Furthermore, the emphasis should be placed on tailoring interventions to the unique needs of individuals based on the stage of injury and the nature of their impairments. Collaborative and multidisciplinary research approaches should be encouraged to address the complex nature of neurorehabilitation. Integrating expertise from neurology, rehabilitation medicine, bioengineering, and other relevant fields will foster a comprehensive understanding of the diverse factors influencing recovery. This collaborative effort can lead to the development of holistic and synergistic interventions that encompass cognitive, motor, and psychosocial aspects of rehabilitation.

In conclusion, the future of neurorehabilitation research should be guided by a commitment to refining and expanding our knowledge base. By addressing gaps in theoretical frameworks, exploring emerging technologies, unraveling the neuroplastic mechanisms, tailoring interventions, and fostering multidisciplinary collaborations, researchers can contribute to the ongoing evolution of neurorehabilitation. Ultimately, the field of neurorehabilitation is rapidly evolving, and there is a need for continued research to develop more effective and personalized treatments for patients with CNS injuries and other neurological disabilities.

## Author contributions

PK: Conceptualization, Data curation, Formal analysis, Investigation, Methodology, Project administration, Supervision, Validation, Visualization, Writing – original draft, Writing – review & editing. AG: Conceptualization, Investigation, Methodology, Project administration, Supervision, Visualization, Writing – original draft, Writing – review & editing. MP: Conceptualization, Investigation, Methodology, Project administration, Supervision, Validation, Visualization, Writing – original draft, Writing – review & editing. AO-P-V: Conceptualization, Investigation, Methodology, Supervision, Validation, Visualization, Writing – original draft, Writing – review & editing. CL-M: Conceptualization, Investigation, Methodology, Project administration, Supervision, Validation, Visualization, Writing – original draft, Writing – review & editing.
